# Ethyl­enedi­ammonium chloride thio­cyanate

**DOI:** 10.1107/S1600536813008830

**Published:** 2013-04-05

**Authors:** Sahel Karoui, Slaheddine Kamoun, François Michaud

**Affiliations:** a Laboratoire de Génie des Matériaux et Environnement, École Nationale d’Ingénieurs de Sfax, BP 1173, Sfax, Tunisia; b Service commun d’analyse par diffraction des rayons X, Université de Brest, 6, Avenue Victor Le Gorgeu, CS 93837, F-29238 Brest cedex 3, France

## Abstract

In the ethyl­enedi­ammonium dication of the title salt, C_2_H_10_N_2_^2+^·Cl^−^·SCN^−^, the N—C—C—N torsion angle is 72.09 (12)°. In the crystal, an extensive three-dimensional hydrogen-bonding network, formed by N—H⋯Cl and N—H⋯N hydrogen bonds, holds all the ions together.

## Related literature

For the crystal structures of related compounds, see: Kamoun *et al.* (1989 ▶); Chen (2009 ▶). For details of the synthesis of thio­cyanic acid, see: Bartlett *et al.* (1969 ▶). For protonic conductivity and dielectric relaxation in ethyl­endi­ammonium salts, see: Karoui *et al.* (2013 ▶).
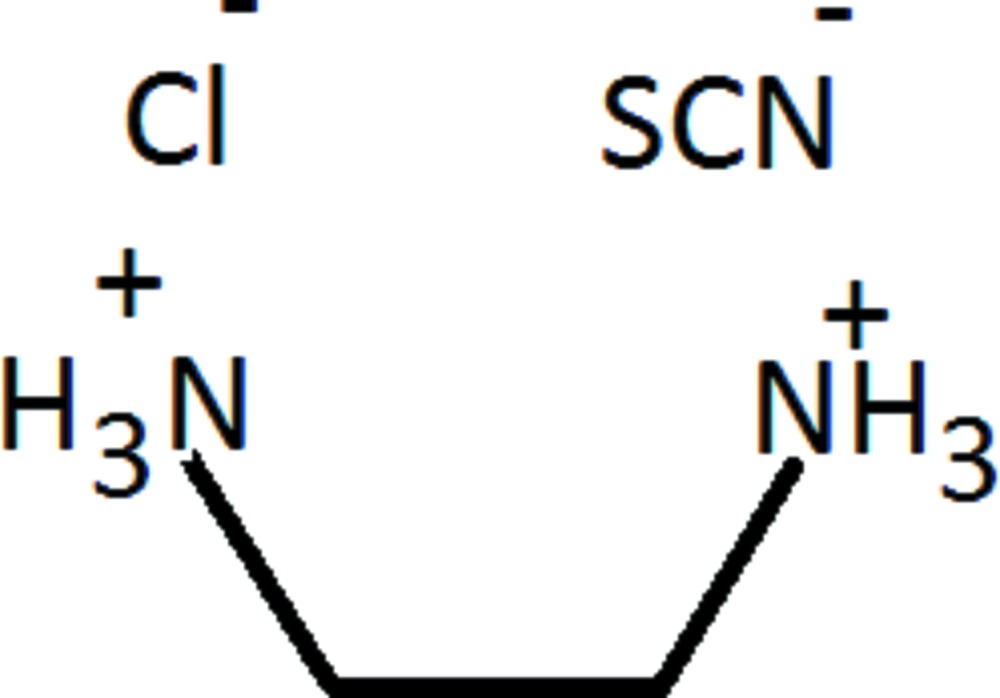


## Experimental

### 

#### Crystal data

C_2_H_10_N_2_^2+^·Cl^−^·SCN^−^
*M* *_r_* = 155.65
Triclinic, 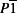

*a* = 6.2726 (2) Å
*b* = 6.3462 (2) Å
*c* = 9.1745 (3) Å
α = 92.436 (3)°
β = 92.193 (3)°
γ = 94.341 (3)°
*V* = 363.52 (2) Å^3^
*Z* = 2
Mo *K*α radiation
μ = 0.72 mm^−1^
*T* = 293 K
0.50 × 0.42 × 0.17 mm

#### Data collection

Agilent Xcalibur (Sapphire2) diffractometer
Absorption correction: multi-scan (*CrysAlis RED*; Agilent, 2012 ▶) *T*_min_ = 0.737, *T*_max_ = 0.887
6396 measured reflections
2189 independent reflections
1947 reflections with *I* > 2σ(*I*)
*R*_int_ = 0.018

#### Refinement

*R*[*F* ^2^ > 2σ(*F* ^2^)] = 0.025
*wR*(*F* ^2^) = 0.072
*S* = 1.08
2189 reflections
74 parameters
H-atom parameters constrained
Δρ_max_ = 0.40 e Å^−3^
Δρ_min_ = −0.25 e Å^−3^

### 

Data collection: *CrysAlis PRO* (Agilent, 2012 ▶); cell refinement: *CrysAlis PRO*; data reduction: *CrysAlis RED* (Agilent, 2012 ▶); program(s) used to solve structure: *SHELXS97* (Sheldrick, 2008 ▶); program(s) used to refine structure: *SHELXL97* (Sheldrick, 2008 ▶); molecular graphics: *DIAMOND* (Brandenburg *et al.*, 1999 ▶) and *Mercury* (Macrae *et al.*, 2008 ▶); software used to prepare material for publication: *WinGX* (Farrugia, 1999 ▶) and *publCIF* (Westrip, 2010 ▶).

## Supplementary Material

Crystal structure: contains datablock(s) I, global. DOI: 10.1107/S1600536813008830/cv5396sup1.cif

Structure factors: contains datablock(s) I. DOI: 10.1107/S1600536813008830/cv5396Isup2.hkl

Supplementary material file. DOI: 10.1107/S1600536813008830/cv5396Isup3.cdx

Additional supplementary materials:  crystallographic information; 3D view; checkCIF report

## Figures and Tables

**Table 1:** Hydrogen-bond geometry (Å, °)

*D*—H⋯*A*	*D*—H	H⋯*A*	*D*⋯*A*	*D*—H⋯*A*
N2—H2*A*⋯Cl1^i^	0.89	2.36	3.1982 (9)	158
N2—H2*B*⋯Cl1^ii^	0.89	2.35	3.2246 (10)	169
N2—H2*C*⋯Cl1^iii^	0.89	2.64	3.3237 (10)	134
N3—H3*C*⋯Cl1^iii^	0.89	2.46	3.2953 (11)	158
N3—H3*A*⋯N1^iv^	0.89	2.03	2.8533 (14)	153
N2—H2*C*⋯Cl1	0.89	2.64	3.3543 (10)	138
N3—H3*B*⋯N1	0.89	2.27	3.1106 (17)	157

Symmetry codes: (i) 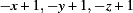; (ii) 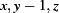; (iii) 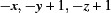; (iv) 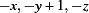.

**Fig. 1.**
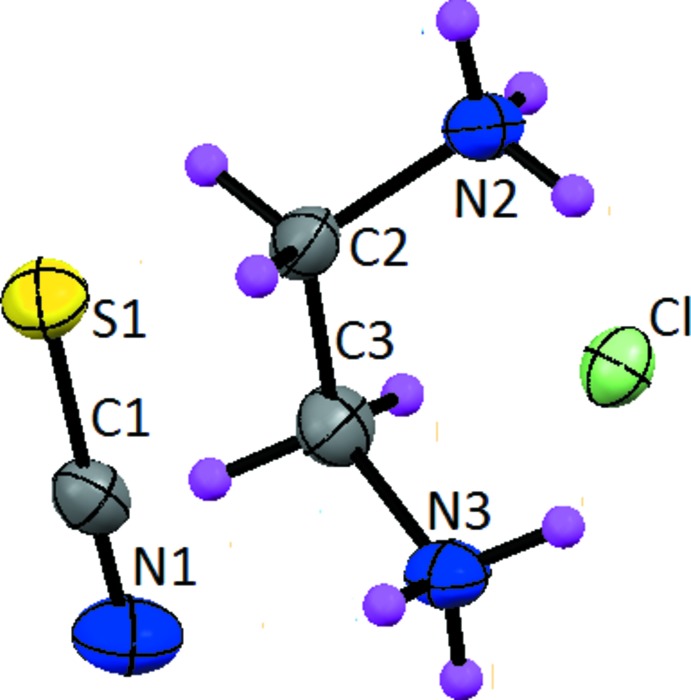
A content of asymmetric unit of (I) showing the atomic-numbering scheme. Displacement ellipsoids are drawn at the 50% probability level. H atoms represented by small spheres of arbitrary radii.

**Fig. 2.**
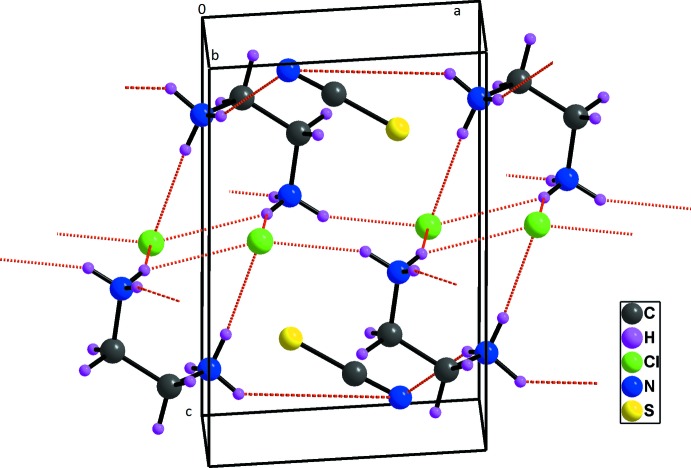
A portion of the crystal packing viewed approximately down the b axis and showing hydrogen bonds as dashed lines.

## supplementary crystallographic information

### Comment

As an extension of our earlier study on protonic conductivity and dielectric relaxation in ethylenediammonium salts (Karoui *et al.*, 2013), we report herein the molecular structure of the title compound, (I), which is a new organic halide-pseudohalide compound.

In (I) (Fig. 1), the asymmetric unit consists of one diprotonated ethylenediammonium cation, one Cl^-^ and one SCN^-^ anions. In this atomic arrangement, the organic group has no internal symmetry. In fact, the mean length of the C—N bonds: 1.4816 (14) Å is lower than that of the C—C bonds:1.5054 (15) Å. The [C_2_H_10_N_2_]^2+^ dication shows an eclipsed conformation with a N–C–C–N torsion angle of 72.09 (12)°. The main geometrical features of this group are similar to that reported for others ethylenediammonium halides (Chen, 2009) and phosphates (Kamoun *et al.*,1989). The thiocyanate ion, present as a monodentate ligand, is almost linear with an angle of 178.48 (11)° and an average C–S and C–N bond lengths of 1.6358 (12) Å and 1.1573 (16) Å, respectively.

In the crystal structure, the ethylenediammonium cations are linked to the chloride and thiocyanate anions by means of five medium N—H···Cl and two weak N—H···N(CS) intermolecular hydrogen bonds (Table 1) to form a three-dimensional network (Fig. 2). The N···Cl and N···N(CS) distances range from 3.1982 (9) to 3.3543 (10) Å and 2.8533 (14) Å to 3.1106 (17) Å, respectively. The sum of Van der Waal's radii of N and Cl, and N and O are 3.3 Å and 2.9 Å, respectively.

### Experimental

The title compound has been obtained as crystalline solid in the reaction of ethylenediamine with an aqueous acidic mixture of hydrochloric acid and thiocyanic acid (1/1 ratio). Thiocyanic acid was prepared using the published procedure (Bartlett *et al.*, 1969). After a slow solvent evaporation yellow crystals suitable for X-ray analysis were obtained.They were washed with diethyl ether and dried over P_2_O_5_.

### Refinement

The H atoms were positioned geometrically (the C—H and N—H bonds were respectively fixed at 0.96 and 0.89), and allowed to ride on their parent atoms, with U_iso_(H) = 1.2 U_eq_(C, N).

### Crystal data

**Table:**

C_2_H_10_N_2_^2+^·Cl^−^·SCN^−^	*F*(000) = 164
*M**_r_* = 155.65	Cell parameters from 3445 reflections
Triclinic, *P*1	*D*_x_ = 1.422 Mg m^−^^3^*D*_m_ = 1.398 Mg m^−^^3^*D*_m_ measured by flotation
Hall symbol: -P 1	Melting point: 443 K
*a* = 6.2726 (2) Å	Mo *K*α radiation, λ = 0.7107 Å
*b* = 6.3462 (2) Å	Cell parameters from 5534 reflections
*c* = 9.1745 (3) Å	θ = 3.2–44.7°
α = 92.436 (3)°	µ = 0.72 mm^−^^1^
β = 92.193 (3)°	*T* = 293 K
γ = 94.341 (3)°	Parallelipipedic, light yellow
*V* = 363.52 (2) Å^3^	0.50 × 0.42 × 0.17 mm
*Z* = 2	

### Data collection

**Table:**

Agilent Xcalibur (Sapphire2) diffractometer	2189 independent reflections
Radiation source: Enhance (Mo) X-ray Source	1947 reflections with *I* > 2σ(*I*)
Graphite monochromator	*R*_int_ = 0.018
Detector resolution: 8.3622 pixels mm^-1^	θ_max_ = 30.5°, θ_min_ = 3.2°
ω scans	*h* = −5→8
Absorption correction: multi-scan (*CrysAlis RED*; Agilent, 2012)	*k* = −9→9
*T*_min_ = 0.737, *T*_max_ = 0.887	*l* = −13→13
6396 measured reflections	

### Refinement

**Table:**

Refinement on *F*^2^	Secondary atom site location: difference Fourier map
Least-squares matrix: full	Hydrogen site location: inferred from neighbouring sites
*R*[*F*^2^ > 2σ(*F*^2^)] = 0.025	H-atom parameters constrained
*wR*(*F*^2^) = 0.072	*w* = 1/[σ^2^(*F*_o_^2^) + (0.0383*P*)^2^ + 0.0716*P*] where *P* = (*F*_o_^2^ + 2*F*_c_^2^)/3
*S* = 1.08	(Δ/σ)_max_ = 0.001
2189 reflections	Δρ_max_ = 0.40 e Å^−^^3^
74 parameters	Δρ_min_ = −0.25 e Å^−^^3^
0 restraints	Extinction correction: *SHELXL97* (Sheldrick, 2008), Fc^*^=kFc[1+0.001xFc^2^λ^3^/sin(2θ)]^-1/4^
0 constraints	Extinction coefficient: 0.294 (15)
Primary atom site location: structure-invariant direct methods	

### Special details

**Table:**

Geometry. All e.s.d.'s (except the e.s.d. in the dihedral angle between two l.s. planes) are estimated using the full covariance matrix. The cell e.s.d.'s are taken into account individually in the estimation of e.s.d.'s in distances, angles and torsion angles; correlations between e.s.d.'s in cell parameters are only used when they are defined by crystal symmetry. An approximate (isotropic) treatment of cell e.s.d.'s is used for estimating e.s.d.'s involving l.s. planes.
Refinement. Refinement of *F*^2^ against ALL reflections. The weighted *R*-factor *wR* and goodness of fit *S* are based on *F*^2^, conventional *R*-factors *R* are based on *F*, with *F* set to zero for negative *F*^2^. The threshold expression of *F*^2^ > σ(*F*^2^) is used only for calculating *R*-factors(gt) *etc*. and is not relevant to the choice of reflections for refinement. *R*-factors based on *F*^2^ are statistically about twice as large as those based on *F*, and *R*- factors based on ALL data will be even larger.

### Fractional atomic coordinates and isotropic or equivalent isotropic displacement parameters (Å^2^)

**Table:**

	*x*	*y*	*z*	*U*_iso_*/*U*_eq_	
Cl1	0.19093 (4)	0.72470 (4)	0.47679 (3)	0.02934 (10)	
S1	0.68519 (5)	0.78130 (5)	0.20850 (3)	0.03649 (10)	
C1	0.45634 (19)	0.73609 (17)	0.11677 (11)	0.0294 (2)	
N1	0.2924 (2)	0.7024 (2)	0.05464 (13)	0.0458 (3)	
N2	0.31015 (15)	0.22026 (15)	0.43178 (10)	0.0305 (2)	
H2A	0.4348	0.2481	0.4811	0.046*	
H2B	0.2590	0.0888	0.4473	0.046*	
H2C	0.2178	0.3109	0.4619	0.046*	
C2	0.34163 (16)	0.24184 (17)	0.27393 (12)	0.0288 (2)	
H2D	0.4449	0.1449	0.2424	0.035*	
H2E	0.4007	0.3844	0.2582	0.035*	
C3	0.13799 (19)	0.19753 (19)	0.18191 (12)	0.0322 (2)	
H3E	0.1733	0.1811	0.0803	0.039*	
H3D	0.0665	0.0651	0.2095	0.039*	
N3	−0.01089 (16)	0.36715 (17)	0.19736 (11)	0.0345 (2)	
H3A	−0.1283	0.3334	0.1412	0.052*	
H3B	0.0525	0.4885	0.1701	0.052*	
H3C	−0.0462	0.3812	0.2901	0.052*	

### Atomic displacement parameters (Å^2^)

**Table:**

	*U*^11^	*U*^22^	*U*^33^	*U*^12^	*U*^13^	*U*^23^
Cl1	0.02482 (13)	0.02877 (14)	0.03390 (15)	−0.00093 (8)	0.00252 (9)	−0.00075 (9)
S1	0.02975 (16)	0.04342 (18)	0.03524 (17)	−0.00037 (11)	−0.00273 (11)	−0.00101 (12)
C1	0.0356 (5)	0.0265 (5)	0.0262 (5)	0.0033 (4)	−0.0003 (4)	0.0020 (3)
N1	0.0446 (6)	0.0482 (6)	0.0434 (6)	0.0040 (5)	−0.0144 (5)	0.0008 (5)
N2	0.0269 (4)	0.0339 (5)	0.0312 (4)	0.0058 (3)	−0.0035 (3)	0.0048 (3)
C2	0.0229 (4)	0.0312 (5)	0.0329 (5)	0.0040 (4)	0.0042 (4)	0.0030 (4)
C3	0.0337 (5)	0.0347 (5)	0.0275 (5)	0.0027 (4)	−0.0009 (4)	−0.0066 (4)
N3	0.0291 (4)	0.0447 (5)	0.0296 (5)	0.0075 (4)	−0.0064 (3)	0.0002 (4)

### Geometric parameters (Å, º)

**Table:**

S1—C1	1.6358 (12)	C2—H2E	0.9700
C1—N1	1.1573 (16)	C3—N3	1.4834 (15)
N2—C2	1.4798 (14)	C3—H3E	0.9700
N2—H2A	0.8900	C3—H3D	0.9700
N2—H2B	0.8900	N3—H3A	0.8900
N2—H2C	0.8900	N3—H3B	0.8900
C2—C3	1.5054 (15)	N3—H3C	0.8900
C2—H2D	0.9700		
			
N1—C1—S1	178.48 (11)	N3—C3—C2	112.98 (9)
C2—N2—H2A	109.5	N3—C3—H3E	109.0
C2—N2—H2B	109.5	C2—C3—H3E	109.0
H2A—N2—H2B	109.5	N3—C3—H3D	109.0
C2—N2—H2C	109.5	C2—C3—H3D	109.0
H2A—N2—H2C	109.5	H3E—C3—H3D	107.8
H2B—N2—H2C	109.5	C3—N3—H3A	109.5
N2—C2—C3	113.06 (9)	C3—N3—H3B	109.5
N2—C2—H2D	109.0	H3A—N3—H3B	109.5
C3—C2—H2D	109.0	C3—N3—H3C	109.5
N2—C2—H2E	109.0	H3A—N3—H3C	109.5
C3—C2—H2E	109.0	H3B—N3—H3C	109.5
H2D—C2—H2E	107.8		
			
N2—C2—C3—N3	72.09 (12)		

### Hydrogen-bond geometry (Å, º)

**Table:**

*D*—H···*A*	*D*—H	H···*A*	*D*···*A*	*D*—H···*A*
N2—H2*A*···Cl1^i^	0.89	2.36	3.1982 (9)	158
N2—H2*B*···Cl1^ii^	0.89	2.35	3.2246 (10)	169
N2—H2*C*···Cl1^iii^	0.89	2.64	3.3237 (10)	134
N3—H3*C*···Cl1^iii^	0.89	2.46	3.2953 (11)	158
N3—H3*A*···N1^iv^	0.89	2.03	2.8533 (14)	153
N2—H2*C*···Cl1	0.89	2.64	3.3543 (10)	138
N3—H3*B*···N1	0.89	2.27	3.1106 (17)	157

Symmetry codes: (i) −*x*+1, −*y*+1, −*z*+1; (ii) *x*, *y*−1, *z*; (iii) −*x*, −*y*+1, −*z*+1; (iv) −*x*, −*y*+1, −*z*.

## References

[bb1] Agilent (2012). *CrysAlis PRO* and *CrysAlis RED* Agilent Technologies, Yarnton, England.

[bb2] Bartlett, H. E., Jurriaanse, A. & De Haas, K. (1969). *Can. J. Chem.* **47**, 16, 2981–2986.

[bb3] Brandenburg, K. & Berndt, M. (1999). *DIAMOND* Crystal Impact GbR, Bonn, Germany.

[bb4] Chen, L.-Z. (2009). *Acta Cryst.* E**65**, o2625. 10.1107/S1600536809039038 PMC2970981 21578241

[bb5] Farrugia, L. J. (1999). *J. Appl. Cryst.* **32**, 837–838.

[bb6] Kamoun, S., Jouini, A., Kamoun, M. & Daoud, A. (1989). *Acta Cryst.* C**45**, 481–482.

[bb7] Karoui, S., Kamoun, S. & Jouini, A. (2013). *J. Solid State Chem.* **197**, 60–68.

[bb8] Macrae, C. F., Bruno, I. J., Chisholm, J. A., Edgington, P. R., McCabe, P., Pidcock, E., Rodriguez-Monge, L., Taylor, R., van de Streek, J. & Wood, P. A. (2008). *J. Appl. Cryst.* **41**, 466–470.

[bb9] Sheldrick, G. M. (2008). *Acta Cryst.* A**64**, 112–122. 10.1107/S0108767307043930 18156677

[bb10] Westrip, S. P. (2010). *J. Appl. Cryst.* **43**, 920–925.

